# Supplementary material for: Empirical methods for controlling false positives and estimating confidence in ChIP-Seq peaks
Source: BMC Bioinformatics. 2008 Dec 5;9:523. doi: 10.1186/1471-2105-9-523 (PMC2628906; doi:10.1186/1471-2105-9-523)
Supplement: Additional File 1 — A variety of html documents from the USeq web site detailing the available applications, their best usage, output file type descriptions, command line menus, etc. [file 1471-2105-9-523-S1.zip › USeqUserGuides/JavaDoc.zip!/JavaDoc/util/apps/gump/Option.html]

Option


|  |  |  |
| --- | --- | --- |
| **Overview** \| **Package** \| **Class** \| **Use** \| **Tree** \| **Deprecated** \| **Index** \| **Help** | |  |
| **PREV CLASS**   NEXT CLASS | **FRAMES**    **NO FRAMES**     **All Classes** |  |
| SUMMARY: NESTED \| FIELD \| CONSTR \| METHOD | DETAIL: FIELD \| CONSTR \| METHOD |  |


---


## util.apps.gump Class Option

```
java.lang.Object
  util.apps.gump.Option
```

---

``` public class Option extends java.lang.Object ```

---

| **Field Summary** | |
| --- | --- |
| `static java.util.regex.Pattern` | `space` |


**Constructor Summary**

`Option(java.lang.String line)`


| **Method Summary** | |
| --- | --- |
| `java.lang.String` | `getDescription()` |
| `javax.swing.JTextArea` | `getDescriptionS()` |
| `java.lang.String` | `getFlag()` |
| `javax.swing.JTextArea` | `getFlagS()` |
| `java.lang.String` | `getNotes()` |
| `static java.util.regex.Pattern` | `getSpace()` |
| `java.lang.String` | `getUserInput()` |
| `javax.swing.JTextField` | `getUserInputS()` |
| `void` | `makeSwingComponents()` |
| `void` | `setDescription(java.lang.String description)` |
| `void` | `setDescriptionS(javax.swing.JTextArea descriptionS)` |
| `void` | `setFlag(java.lang.String flag)` |
| `void` | `setFlagS(javax.swing.JTextArea flagS)` |
| `void` | `setNotes(java.lang.String notes)` |
| `void` | `setUserInput(java.lang.String userInput)` |
| `void` | `setUserInputS(javax.swing.JTextField userInputS)` |
| `java.lang.String` | `toString()` |

**Methods inherited from class java.lang.Object**

`equals, getClass, hashCode, notify, notifyAll, wait, wait, wait`

**Field Detail**

### space

```
public static final java.util.regex.Pattern space
```


**Constructor Detail**

### Option

```
public Option(java.lang.String line)
```


**Method Detail**

### makeSwingComponents

```
public void makeSwingComponents()
```

---


### toString

```
public java.lang.String toString()
```

:   **Overrides:**: `toString` in class `java.lang.Object`

---


### getFlag

```
public java.lang.String getFlag()
```

---


### setFlag

```
public void setFlag(java.lang.String flag)
```

---


### getNotes

```
public java.lang.String getNotes()
```

---


### setNotes

```
public void setNotes(java.lang.String notes)
```

---


### getUserInput

```
public java.lang.String getUserInput()
```

---


### setUserInput

```
public void setUserInput(java.lang.String userInput)
```

---


### getSpace

```
public static java.util.regex.Pattern getSpace()
```

---


### getDescription

```
public java.lang.String getDescription()
```

---


### setDescription

```
public void setDescription(java.lang.String description)
```

---


### getUserInputS

```
public javax.swing.JTextField getUserInputS()
```

---


### setUserInputS

```
public void setUserInputS(javax.swing.JTextField userInputS)
```

---


### getFlagS

```
public javax.swing.JTextArea getFlagS()
```

---


### setFlagS

```
public void setFlagS(javax.swing.JTextArea flagS)
```

---


### getDescriptionS

```
public javax.swing.JTextArea getDescriptionS()
```

---


### setDescriptionS

```
public void setDescriptionS(javax.swing.JTextArea descriptionS)
```


---


|  |  |  |
| --- | --- | --- |
| **Overview** \| **Package** \| **Class** \| **Use** \| **Tree** \| **Deprecated** \| **Index** \| **Help** | |  |
| **PREV CLASS**   NEXT CLASS | **FRAMES**    **NO FRAMES**     **All Classes** |  |
| SUMMARY: NESTED \| FIELD \| CONSTR \| METHOD | DETAIL: FIELD \| CONSTR \| METHOD |  |


---
